# Exploring machine learning classification for community based health insurance enrollment in Ethiopia

**DOI:** 10.3389/fpubh.2025.1549210

**Published:** 2025-07-18

**Authors:** Seyifemickael Amare Yilema, Yegnanew A. Shiferaw, Yikeber Abebaw Moyehodie, Setegn Muche Fenta, Denekew Bitew Belay, Haile Mekonnen Fenta, Teshager Zerihun Nigussie, Ding-Geng Chen

**Affiliations:** ^1^Department of Statistics, Debre Tabor University, Debre Tabor, Ethiopia; ^2^Department of Statistics, University of Pretoria, Pretoria, South Africa; ^3^Department of Statistics, University of Johannesburg, Johannesburg, South Africa; ^4^Department of Statistics, College of Science, Bahir Dar University, Bahir Dar, Ethiopia; ^5^Center for Environmental and Respiratory Health Research (CERH), Research Unit of Population Health, University of Oulu, Oulu, Finland; ^6^College of Health Solutions, Arizona State University, Phoenix, AZ, United States

**Keywords:** machine learning, health insurance, random forest, accuracy, Ethiopia

## Abstract

**Background:**

Community-based health insurance (CBHI) is a vital tool for achieving universal health coverage (UHC), a key global health priority outlined in the sustainable development goals (SDGs). Sub-Saharan Africa continues to face challenges in achieving UHC and protecting individuals from the financial burden of disease. As a result, CBHI has become popular in low- and middle-income countries, including Ethiopia. Therefore, this study aimed to identify the ML algorithm with the best predictive accuracy for CBHI enrollment and to determine the most influential predictors among the dataset.

**Methods:**

The 2019 Ethiopian Mini Demographic and Health Survey (EMDHS) data were used. The CBHI were predicted using seven machine learning models: linear discriminant analysis (LDA), support vector machine with radial basis function (SVM), k-nearest neighbors (KNN), classification and regression tree (CART), and random forest (RF). Receiver operating characteristic curves and other metrics were used to evaluate each model’s accuracy.

**Results:**

The RF algorithm was determined to be the best machine learning model based on different performance assessments. The result indicates that age, wealth index, household members, and land usage all significantly affect CBHI in Ethiopia.

**Conclusion:**

This study found that RF machine learning models could improve the ability to classify CBHI in Ethiopia with high accuracy. Age, wealth index, household members, and land utilization are some of the most significant variables associated with CBHI that were determined by feature importance. The results of the study can help health professionals and policymakers create focused strategies to improve CBHI enrollment in Ethiopia.

## Introduction

Community-based health insurance (CBHI) is a health insurance plan that provides improved access to medical care and financial security against the high expense of illness (1). It is a voluntary, non-profit medical insurance, generally established at the community level, particularly targeting individuals working in the informal sector (2–4). It is a risk-sharing technique to spread healthcare costs among families by allowing cross-subsidies from high-income households to disadvantaged populations (4).

Regardless of living standards, everyone must have enough access to the required medical care without facing financial hardship. Universal health coverage (UHC) aims to ensure individuals get access to the high-quality healthcare when they fall ill without suffering financial difficulties (4, 5). A robust health system with reliable financing is needed to achieve UHC (6). However, poor health care financing is still a major barrier to the low-income society’s health services utilization. To reduce financial obstacles to the use of health services, several countries established various insurance programs (2, 7).

Globally, over 150 million people suffer financial catastrophes due to out-of-pocket medical expenses on health services (8). CBHI has become a feasible alternative for financing healthcare services in developing countries due to the high cost and the impact of out-of-pocket expenses on households in developing countries, many families face financial strain that can hinder their access to essential services such as healthcare, education, and basic necessities. This can lead to increased poverty levels, as households may struggle to afford necessary treatments or educational opportunities, ultimately affecting their quality of life and economic stability. CBHI programs were introduced as a risk-sharing mechanism for rural communities, self-employed and unemployed contracted informal workers, and those with poorer economies in many low- and middle-income nations, including Ethiopia (2, 7, 9).

In most African countries, more than 40% of their overall medical expenses came from out-of-pocket spending, which left the healthcare system low on funding (2, 4, 6). In sub-Saharan African countries, out-of-pocket expenditure can be a significant obstacle to receiving quality medical treatment. It has been recommended that low-income nations increase their healthcare spending to around 4.6% of their gross domestic product (GDP) by 2030 to meet the sustainable development goal (SDG) pertaining to health (9, 10). Furthermore, projections suggest that to achieve progress toward UHC (11, 12), government health spending in these countries must equal at least 5% of the GDP. However, government spending on health care has mostly stayed below 2% of GDP in many SSA nations, including Ethiopia (10, 12, 13). The majority of SSA nations have experienced financial issues in paying healthcare (9). CBHI has become an effective risk-pooling method to offer populations some financial security (4).

However, the evaluation of CBHI shows that, quite apart from a few successful experiences with an example of schemes suffer from persistently low membership that may be related to lower socioeconomic status, poor health care quality, lack of benefit from the scheme, lack of trust in the management of the scheme, and dissatisfaction with the services provided by the scheme (2).

Ethiopia enacted the CBHI policy in 2011 to enhance the country’s health care finance system (2, 7, 14). The Ethiopian CBHI program is characterized as a government-run project with community participation in the design, implementation, and oversight of the program. Members’ premium contributions represent the majority of the scheme’s funding, with the central government contributing about 25% of the overall premium subsidy (15, 16). In spite of significant efforts to increase access to modern health services over the past years, Ethiopians continue to use medical services using the CBHI methods though it was low rates (15, 16).

Predicting the frequency or probability of insurance enrollment in a specific accident or scheme becomes challenging due to the imbalanced dataset since the number of non-enrollments is significantly higher than enrollments. This imbalance might have happened due to the government policy enforcement capacity on awareness creation about the importance of CBHI to household health improvement and financial coverage when they fall ill (17). Traditional classification models, such as logistic regression, have a limited ability to predict the enrollment of households to CBHI. Therefore, employing machine learning models for predicting CBHI enrollment status provides accurate predictions. Machine learning (ML) is concerned with computer programs/algorithms that improve their performance automatically via experience (18). ML is a subfield of artificial intelligence that is built on the premise that a machine may learn from data, find patterns, and make decisions with little or no human intervention and without being explicitly programmed (19–21). It is a robust method that combines artificial intelligence with statistical learning. When tackling categorization challenges, ML algorithms have demonstrated higher prediction capabilities when compared to traditional applied medical research (21, 22). The prediction accuracies of ML models are not consistent for imbalanced insurance data, and the performances of several classification ML models were compared using model performance metrics. In addition, methods of sampling procedures are affecting the accuracy of CBHI enrollment status, and we used synthetic minority over-sampling techniques (SMOTE) to optimize the minority classes.

From the best of our knowledge, there is a gap in employing a machine learning approach to handle classification imbalance in CBHI enrolment in Ethiopia. This research is then aimed to identify ML algorithms for predicting CBHI using SMOTE resampling approach for imbalanced insurance data, and then narrowing the existing literature gaps.

## Methods and materials

### Data sources and study variables

This study used data from the 2019 Ethiopian Mini Demographic and Health Survey (EMDHS). After obtaining permission through an online request and describing the purpose of the study, the data were released online via the website.^1^ The sample for the 2019 EMDHS was selected using a two-stage stratified cluster sampling design. The nine regions and two city administrations were classified as urban and rural areas, and divided into 21 sampling strata. In the first stage, 305 EAs were selected independently using a probability proportional to EA size. The second step of the selection procedure, on average 30 households per EA were systematically selected with equal probability from the freshly constructed household lists in the selected EAs. Substitutions or modifications to the preselected homes were not permitted during the implementation period to prevent bias.

### Study variables

#### Outcome variable

The outcome variable of the study was CBHI enrollment, and it was categorized as either “Yes” (labeled as 1) if enrolled for CBHI or “No” (labeled as 0) if the household did not enroll for CBHI (23, 24).

#### Independent variables

The predictor variables were the sex of the head of household (Male, Female), having a mobile telephone (No, Yes), having land for agriculture (No, Yes), owning livestock herds, or farm animals (No, Yes), having a radio (No, Yes), television (Yes, No), watching any media (Yes, No) wealth index (Poorest, Poorer, Middle, Richer, Richest), receiving cash for food from the safety net program (No, Yes), Education level of household head (No Education, Primary, Secondary and above), age of household heads (15–34 ages, 35–54 ages, 55–74 ages, ≥75 ages), number of household members (1–3 members, 4–6 members, 7–9 members, ≥10 members), number of children 5 and under (No child, 1–2 children, ≥3 children), residence (urban, rural) (23).

### Data pre-processing

The data pre-processing reduces prediction errors and improves the efficiency of the machine learning model. However, a careful selection of data pre-processing techniques is required to significantly influence the final prediction, which might negatively impact the prediction performance of machine learning methods (25). We excluded non-respondents, as 8,663 (98.51%) of the 8,794 individuals selected for the interview provided satisfactory responses to the questions on health insurance enrollment.

The training and testing data division ratio greatly influences the predictive abilities of the machine learning models. The training subset of the data is used to fit the proposed model, and an evaluation of the adequacy of the model. The testing dataset also called the validation set, is used to criticize the out-of-sample predictive capability of the models (25). There are many ways to split the sample data into training and test sets. The most commonly used training and testing data-splitting ratios are between 90/10, 80/20, and 70/30. A simulation study by Nguyen et al. (25) revealed that 70/30 data splitting offers the best performance for classification machine learning models. Therefore, to evaluate model performance, the dataset was partitioned into a training set (70%) and a testing set (30%) based on the best-performing model (22).

### Statistical machine learning analysis

CBHI predictions were made using machine learning algorithms such as Logistic regression (LR), linear discriminant analysis (LDA), support vector machine with radial basis function (SVM), k-nearest neighbors (KNN), classification and regression trees (CART), and random forest (RF).

### Logistic regression (LR)

Logistic regression (LR) is a popular traditional model that often relies on strict assumptions such as linearity and independence of predictors and was used as a baseline model machine learning technique (26, 27). The categorical dependent variable is predicted using a particular collection of independent factors. Modeling the probability of a specific class or outcome occurring given the values of the independent variables is the main goal. The “best fitting model” in logistic regression is made up of the best parameters that describe the correlation between the log-odds of the dependent variable and the independent variables.

Linear discriminant analysis (LDA): LDA is a dimensionality reduction technique. In machine learning and pattern classification applications, it is a preprocessing phase. In order to escape the dimensionality curse and save money and resources, LDA plans features from higher higher-dimensional space to a lower-dimensional space. LDA is a supervised classification method that is used in the building of effective machine learning models. This type of dimensionality reduction is employed in fields like image identification and predictive analysis (28).

Support vector machine with the radial basis function (SVM): support vector machine (SVM) is used to successfully build nonlinear classifiers. SVMs are part of the class of kernel methods, which are maximum margin classifiers, and attempt to maximize the distance from support vectors to a hyperplane for generating the best decision boundary. Radial basis functions are used within SVM to train machine learning models (28, 29).

K-nearest neighbors (KNN): For each piece of training data, the classifier calculates the Euclidean distance between fresh data points. The K entries closest to the new data point are then chosen. The new data point class label is based on the label having the highest frequency across K entries. As a result, the new data point will be categorized as non-uptake if non-uptake is the most prevalent and vice versa (18, 28).

Classification and regression trees (CART): CART is machine learning method that is used to construct prediction models from datasets. The ML models are obtained by recursively partitioning the data space and fitting a simple prediction model within each partition. The CART algorithm is one of the ML algorithms, which is a classification algorithm required to build a decision tree. It is an essential ML algorithm and provides a wide variety of use cases (18, 22).

Random forest (RF): RF is the most powerful ensemble-based method (30) widely utilized in regression and classification problems. Random forests are a tree predictor combination in which each tree is reliant on the values of a random vector that are sampled independently and with the same distribution for all trees in the forest (30, 31). It develops a powerful prediction algorithm for identifying community-based health insurance coverage at the community level, which can then be used to address various real-world health concerns (18, 28). These ML model-based algorithms are compared, and the best model is chosen based on the model evaluation criteria.

Machine learning model performance evaluation: Accuracy, precision, recall, specificity, F1 score, and AUROC were accuracy metrics used to evaluate the performance of the ML predictive models (18). Each machine learning algorithm’s chance of properly classifying a random sample is explained by the aggregated value provided by the AUC. The AUC of the receiver characteristics curve (ROC), averaged across ten cross-validation folds (ten repetitions), divides the original sample into ten disjoint subsets, uses nine of those subsets for training, and then forecasts the remaining subset (18, 32–38).

### Performance measures

A diagnosis of class imbalance is made by looking at the distribution of the outcome variable, which is health insurance enrollment. The minority class’s percentage of people with insurance enrollment was much lower than the majority class’s percentage of people without insurance (39, 40). Various literatures classify the degree (severity) of class imbalance based on the minority proportion into three categories: mild (20–40%), moderate (1–20%), and severe (<1%) (40, 41). Descriptive statistics from the current study show that the majority of respondents (79.85%) do not have health insurance, while the minority of respondents (20.15%) enroll in health insurance. This demonstrates how class disparity can produce skewed models that favor the majority class and produce inaccurate assessment measures (42). Specifically, the minority class constituted around 20% of the dataset, indicating a substantial imbalance that could negatively bias the performance of standard classifiers toward the majority class.

To address this, we implemented the synthetic minority oversampling technique (SMOTE), which generates synthetic samples of the minority class by interpolating between existing minority class instances. We used internal 10-fold cross-validation to keep from overfitting while also making sure that model performance remains independent of a single train-test split. This method gives us a more thorough assessment of the model’s prediction performance by enabling us to evaluate its stability and generalizability over multiple data subsets. This technique helps improve the classifier’s ability to learn the decision boundary between classes, which in turn enhances performance metrics such as AUC, accuracy, precision, recall and F1-score for the minority class crucial in health-related applications like health insurance prediction, where correctly identifying positive cases (enrolled insurance) is essential.

The basic model performance evaluation metrics are derived from the confusion matrix as follows.


$$\text{Accuracy}=\frac{\mathrm{TP}+\mathrm{TN}}{\mathrm{TP}+\mathrm{TN}+\mathrm{FP}+\mathrm{FN}}$$

$$\text{Precision}=\frac{\mathrm{TP}}{\mathrm{TP}+\mathrm{FP}}$$

$$\text{Recall}=\frac{\mathrm{TP}}{\mathrm{TP}+\mathrm{FN}}$$

$$F_{1}-\text{score}=\frac{2*\text{Precision}\times \text{Recall}}{\text{Precision}+\text{Recall}}$$

$$\text{Specificity}=\frac{\mathrm{TN}}{\mathrm{TN}+\mathrm{FP}}$$

where TP,TN,FP,FN refer to true positive, true negative, false positive, and false negative, respectively. For this study, Python 3.12.4 is used alongside the Jupyter notebook environment, which offers a web-based interface for producing and sharing the results of computations for ML SMOTE methods (43).

## Results and discussion

### CBHI enrolment and its socio-demographic features

A total of 8,663 study participants were included in the analysis. Respondents with no formal education had a greater prevalence of CBHI enrolment (23.1%) than those with secondary and higher education (11.9%). Compared to respondents with higher wealth index (18.5%), middle-class respondents (33.9%) were more likely to be enrolled in CBHI. The CBHI enrolment rate was higher among rural respondents (23.9%) than among urban respondents (11.7%). When compared to other regions, respondents from the Tigray region had higher CBHI enrolment (49.2%). Furthermore, variables that had significant association with CBHI enrollment status were used to -train machine learning algorithms to predict the CBHI enrollment status of respondents using the training dataset (Table 1).

**Table 1:** Summary statistics of CBHI enrolment for selected variables included in the analysis.

Variables	Categories	CBHI enrollment		*χ*^2^ test statistic	*p-*values
		No (%)	Yes (%)		
Educational attainment	No education	77.0	23.0	99.13	<0.001
	Primary	78.7	21.3		
	Secondary and above	88.1	11.9		
Income	Poor	83.1	16.9	178.86	<0.001
	Middle	66.1	33.9		
	Rich	81.5	18.5		
Age of household heads	15–34 ages	86.7	13.3	143.177	<0.001
	35–54 ages	77.5	22.5		
	55–74 ages	73.7	26.3		
	≥ 75 ages	75.8	24.2		
Household size	1–3	83.4	16.6	50.39	<0.001
	4–6	76.7	23.3		
	7–10	79.5	20.5		
	11 and above	84.9	15.1		
Owns land usable for agriculture	No	88.4	11.6	370.32	<0.001
	Yes	71.8	28.2		
Has mobile telephone	No	79.7	20.3	0.084	3.96
	Yes	79.9	20.1		
Sex of head of household	Male	78.5	21.5	25.50	<0.001
	Female	83.4	16.6		
Has television	No	77.7	22.3	81.77	<0.001
	Yes	87.0	13.0		
Has radio	No	79.6	20.4	0.836	0.188
	Yes	80.5	19.5		
Residence	Urban	88.3	11.7	168.32	<0.001
	Rural	76.1	23.9		
Owns livestock, herds or farm animals	No	88.0	12.0	235.86	<0.001
	Yes	74.5	25.5		
Has bank account	No	80.9	19.1	8.07	0.002
	Yes	78.4	21.6		
Receiving cash of food from the safety Net Program	No	81.7	18.3	87.95	<0.001
	Yes	70.9	29.1		
Region	Tigray	50.8	49.2	1762.98	<0.001
	Afar	97.0	3.0		
	Amhara	42.1	57.9		
	Oromia	79.2	20.8		
	Somali	96.5	3.5		
	Benishangul-Gumuz	89.6	10.4		
	SNNPR	79.0	21.0		
	Gambela	92.6	7.4		
	Harari	87.9	12.1		
	Addis Ababa	88.0	12.0		
	Dire Dawa	93.9	6.1		

### Prevalence of CBHI enrollment in Ethiopia

**Figure 1:**
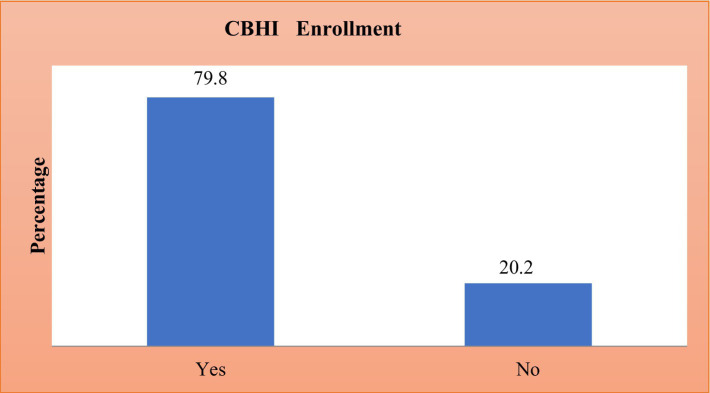
Prevalence of CBHI enrollment in Ethiopia based on the 2019 survey data.

The prevalence of CBHI enrolment in Ethiopia was presented in Figure 1. In Ethiopia, 20.15% of people were enrolled in the CBHI scheme (95% CI: 19.31, 21.025) (Figure 1).

### Feature importance using the RF algorithm

**Figure 2:**
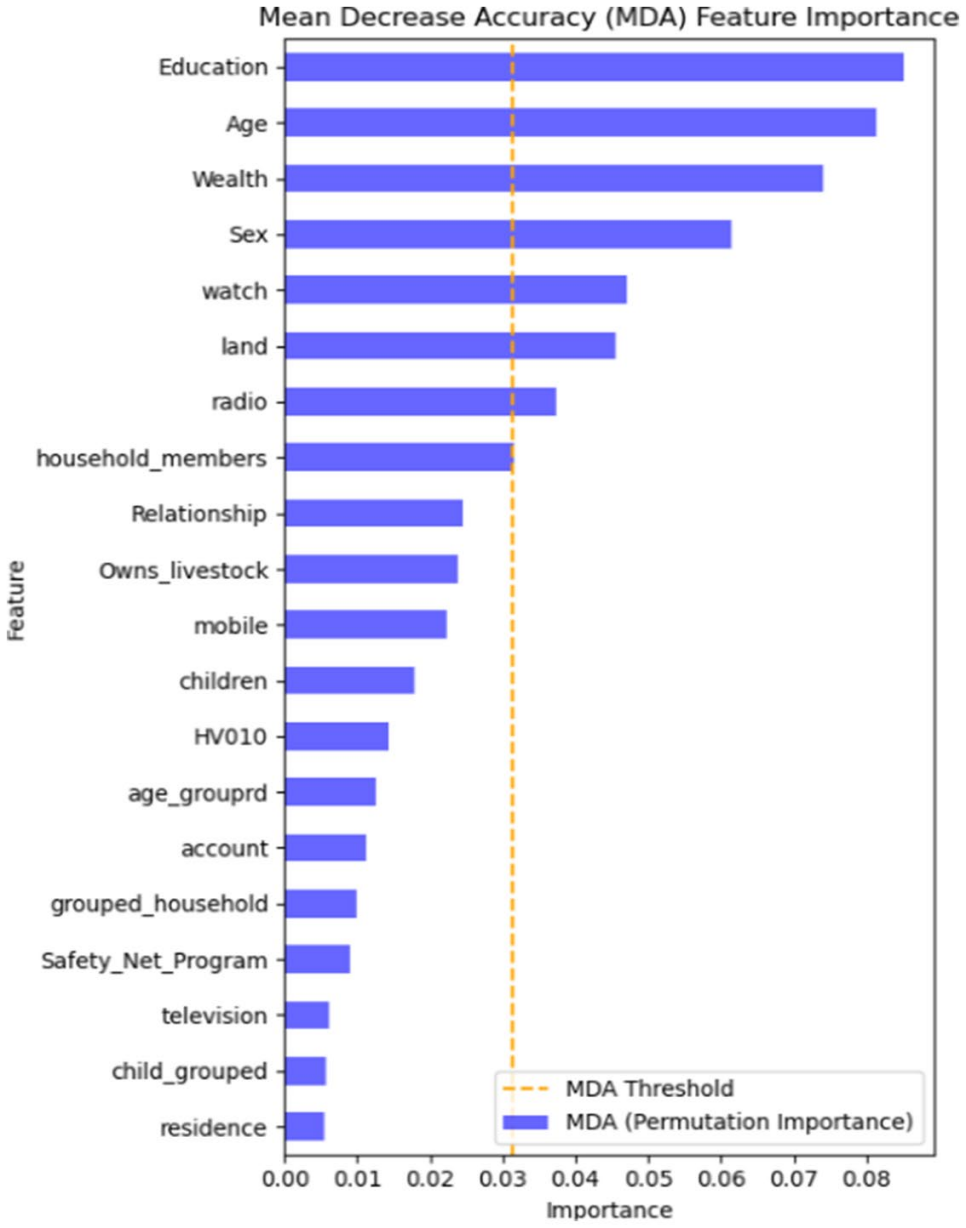
Feature importance using MDA in the RF algorithm.

Finding the most crucial factors in the data is crucial for machine learning classification. There are several ways to do this, but in this study, we employed the information gain rank approach to determine the key variables linked to CBHI. According to the mean decreasing accuracy (MDA) feature importance, the top variables that above the MDA threshold values are crucial for the predictions made by the machine learning model (Figure 2). The findings, presented in Figure 2, suggest that educational level, age, wealth, sex, and land usage are the top important features that most influence CBHI enrollment in the machine learning model prediction.

### Model building and evaluations

To evaluate model performance and select the most accurate predictive models, we used internal 10-fold cross-validation, which provides a robust estimate of model generalizability. The LR, CART, LDA, KNN, and RF models had mean accuracy higher than 80%, which are the best models. However, the accuracy of the RF model was greater than the other models, with a mean accuracy of 84.80. Therefore, RF is the best model for predicting community-based insurance enrollment in Ethiopia (Table 2).

**Table 2:** ML models’ accuracy metrics.

Model	Accuracy					Kappa				
	Min	1^st^ Q	Mean	3^rd^ Q	Max	Min	1^st^ Q	Mean	3^rd^ Q	Max
CART	82.56	83.39	84.07	84.49	86.03	34.24	36.95	40.11	43.18	47.85
LDA	80.02	81.08	81.74	82.42	83.62	17.80	22.14	25.98	29.15	34.21
SVM	66.23	72.72	76.79	80.52	85.72	17.40	37.78	40.03	45.91	51.81
KNN	81.87	83.01	83.60	84.08	85.90	35.69	38.04	41.50	43.49	50.83
RF	83.35	84.3	84.80	85.20	86.61	40.80	44.40	46.15	47.90	52.10
LR	69.30	71.0	72.50	74.25	76.12	17.40	19.80	23.10	25.62	29.50

**Figure 3:**
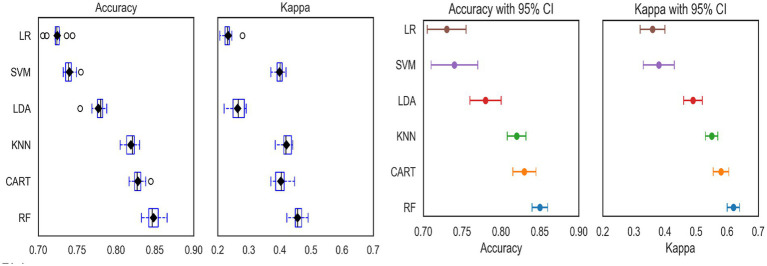
The box-whisker (left) and dot plots (right) for accuracy and kappa statistics for accuracy of different machine learning models.

The box-whisker and dot plots for accuracy and kappa statistics are also presented in Figure 3. It is worth noting that the boxes are arranged in descending order of mean accuracy. The dots in the box and whisker plots are the mean accuracy and kappa, which contain the middle values of the results. The dot plots in Figure 3 are essential to show all ML algorithms’ mean accuracy and 95% confidence intervals. In both plots, the accuracy and kappa statistics of RF are better than the other ML classifiers. Thus, we confirmed that the RF algorithm best predicts CBHI analysis (Figure 3).

Table 3 present the accuracy, precision, recall, and F1-scores of each machine learning algorithm following the application of the SMOTE technique. The RF model was superior to the other machine learning prediction models based on the model’s prediction accuracy. The accuracy of RF model for precision, recall, F1-score, and AUC are 78.8, 82.6, 80.6, and 88.2%, respectively (Table 3). For models where precision and recall are equally significant, the F1-score, which combines the two is significant. The better the classifier, the closer the F1-score is to one. The F1-Scores for RF, CART, KNN, and LR in the current study are 0.806, 0.755, 0.788, and 0.669, respectively, indicating that RF is the best classifier when compared to the other approaches. Consequently, it can be concluded that the RF model performs best in terms of both predictive power and balance.

**Table 3:** Performance evaluation of the selected ML algorithms for CBHI prediction.

Classifier	Brier loss	Log loss	AUC	Precision	Accuracy	Recall	F1-score
RF	0.141	0.479	0.882	0.788	0.802	0.826	0.806
CART	0.252	0.874	0.747	0.719	0.743	0.794	0.755
SVM	0.214	0.618	0.720	0.636	0.656	0.728	0.679
NB	0.267	0.049	0.726	0.637	0.659	0.734	0.682
KNN	0.185	0.442	0.825	0.702	0.759	0.899	0.788
LR	0.206	5.599	0.714	0.620	0.651	0.710	0.669

**Figure 4:**
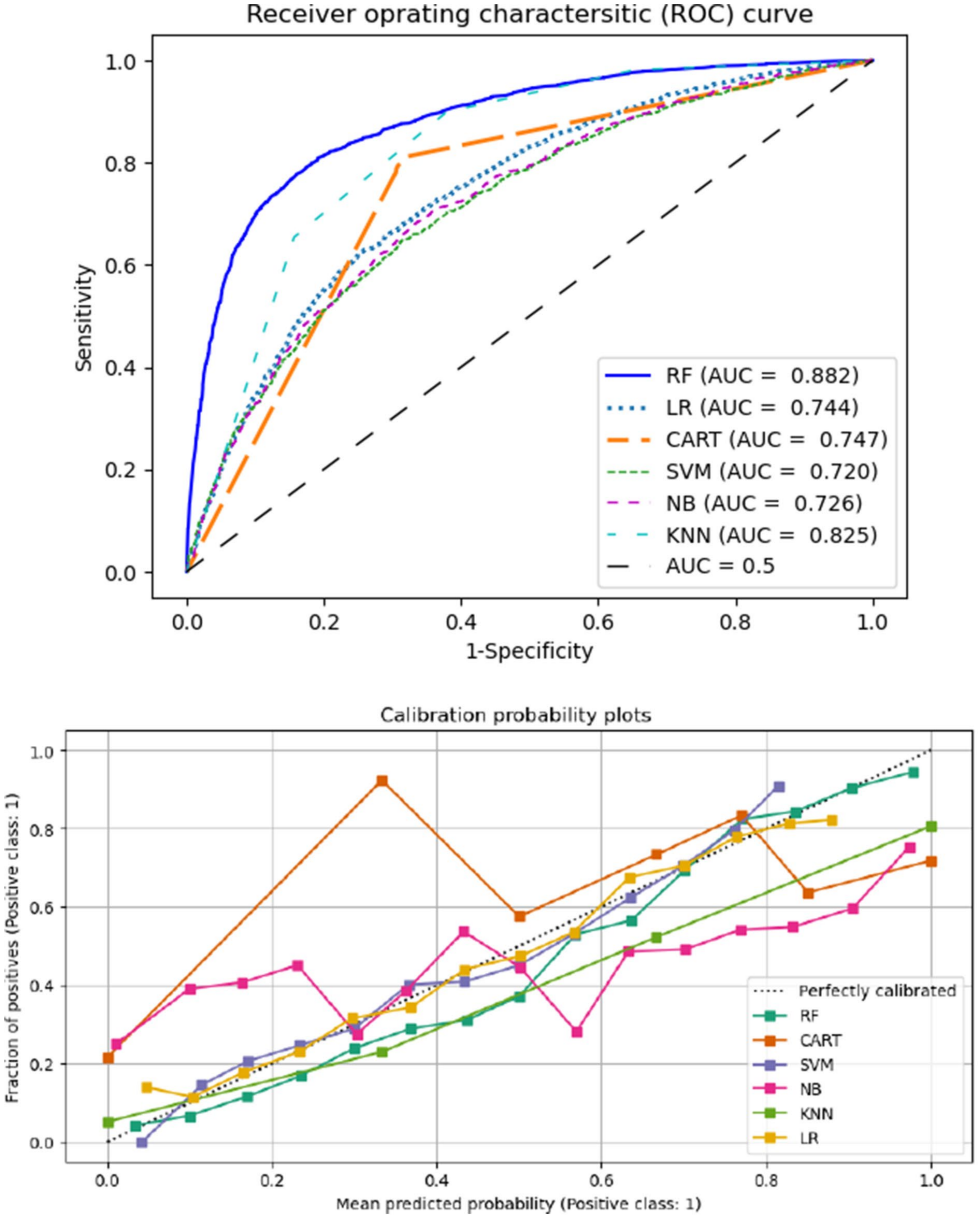
Areas under the receiver operating characteristics curve (AUCs) (top) and the calibration probabilities (bottom).

The trade-off between true positive and false positive rates at different classification thresholds is represented graphically by the ROC curve. The ROC curve is especially helpful for threshold setting, model comparison, and situations involving imbalanced classes. It is a useful tool for assessing the overall performance of a classification model because of its threshold insensitivity and visual depiction. The calibration probability plot and ROC curve for several machine learning techniques are displayed in Figure 4. As a result, following resampling with SMOTE, the RF model had higher performance and has largest AUC values compared to other ML models included in this study. Furthermore, after the mean projected probability of 0.8, the RF model is almost exactly aligned with the 45-degree line, indicating that it performed better than other ML algorithms.

## Discussion

This paper compares several algorithms to identify the most effective ones for analyzing CBHI survey data. The binary outcome variable, community-level health insurance status, from the 2019 EMDHS was used for training and validating the models. The most appropriate performance metrics and visual data representations were chosen from those available. The main strength of this paper was twofold (i) it identified the essential variables via the best ML algorithm. (ii) This is the first Ethiopian study to predict health insurance survey data using ML algorithms (CART, LDA, SVM, KNN, and RF). This study aims to compare and evaluate the performance of various machine learning (ML) algorithms by considering the impact of a 70/30 training–testing split ratio on the prediction of CBHI classification. Common statistical performance metrics such as accuracy, Cohen’s kappa, and various diagnostic plots were used to assess the predictive power of the ML algorithms under this validation scheme.

The comparisons of different ML model algorithm for the predictive capacity presented by the different graphs (box-whisker plots, dot plots, and ROC curve) and algorithmic performance measurements (31, 44). It is worth noting that, although having the lowest classification accuracy when compared to the RF and CART algorithms, the SVM with radial kernel is a very interpretable estimated classifier (31). The AUC under the ROC curve the ML algorithms are 74.7 for CART, 72.00 for SVM, 82.50 for KNN, 71.4 for LR, and 88.2 for RF, which shows RF is preferable to the other ML models. In addition, the ML algorithms SVM, CART, KNN, and RF outperform the conventional LR methods in terms of precision, recall, and F1-score. This demonstrates that ML techniques perform better than the conventional normally LR model. Furthermore, the accuracy, precision, and F1-score performance metrics of the RF model are better the other algorithms, which confirms the RF model performs relatively better in ML classifications of health insurance enrollment. The current finding is consistent with other studies conducted in Ethiopia for under five children malnutrition, and renal graft failures (22, 40). Other studies conducted in United Nation for an educational virtual reality environment findings show that RF provide better accuracy (98%) compared to SVM model (45). According to a comprehensive review conducted in 17 papers evaluating several supervised machine learning algorithms for disease prediction, RF has the greatest accuracy in 9 of them (53%) (31). Therefore, this shows that most study findings are in line with the current research findings that revealed RF is better performance accuracy than other alternative ML algorithms (31). In contrast from among 17 papers, RF algorithm is not the most superior for 47% of them. In contrast, 47% of the 17 reviewed articles did not use the RF algorithm as the best-performing model (31).

This study is focusing on identify the most important features for predicting ML algorithms. The important features in the current study are selected based on the RF algorithm for CBHI enrollment. The most important features are education, age, wealth, and land usage for CBHI data. Various studies have been conducted on different aspects of the ML algorithms for feature selection and confirmed that wealth and age are among the top important features (21, 22). Moreover, the variables of study are consistent with the conventional generalized linear model, which indicates that the most significant features for CBHI were maternal education, age, wealth, sex and land usage, according to the chosen machine learning technique (7, 23, 46).

Maternal education emerged as a vital predictor, indicating that policies that empower women through education may have a long-term favorable impact on health insurance results. Other studies’ findings are consistent with the findings from this study, such as the more educated respondents can have a high chance of enrolling in the CBHI scheme (23, 47). The age of respondents for health insurance enrollment was particularly important, indicating that age-specific interventions can be better envisioned. Furthermore, the wealth index emphasizes the importance of specific health insurance enrollment support programs for respondents from low-income households, which aligns with larger poverty reduction goals. Another key predictor was rural living, stressing the need of allocating resources to rural communities with inadequate access to healthcare services. Finally, the gender of respondents enrolling in health insurance shows that gender-specific concerns should be considered when developing health policy measures (14, 48, 49).

## Conclusion

This study’s primary goal was to assess and compare the effectiveness of several ML methods to predict the CBHI enrollment status of households in Ethiopia using the 2019 mini EDHS. To assess the classification power of the ML algorithms under various testing and training ratios, different statistical metrics, including accuracy and area under the curve, were used. A model with better performance had higher accuracy, and results show that machine learning models can classify the CBHI with high accuracy. The RF was the best model with an accuracy and AUC of 80.2 and 88.2%, respectively. Maternal education, age of respondents, wealth index, sex, media, and land utilization are some of the most significant variables for the prediction of CBHI enrolment status of households in Ethiopia. Governments and stakeholders on education should prioritize expanding female education since educated women more likely to engage in the CBHI scheme. In addition, governments and policymakers tiered premium structures to reduce financial constraints for lower income households making health insurance more equitable and accessible to all.

## Data Availability

The datasets presented in this study can be found in online repositories. The names of the repository/repositories and accession number(s) can be found in the article/supplementary material.

## Footnotes

^1^ https://www.dhsprogram.com/data
